# EEG data collection using visual evoked, steady state visual evoked and motor image task, designed to brain computer interfaces (BCI) development

**DOI:** 10.1016/j.dib.2019.103871

**Published:** 2019-03-22

**Authors:** S.M. Fernandez-Fraga, M.A. Aceves-Fernandez, J.C. Pedraza-Ortega

**Affiliations:** a Tecnólogico Nacional de México/Instituto Tecnológico de Querétaro Mexico; b Universidad Autónoma de Querétaro Mexico

## Abstract

A set of electroencephalogram (EEG) data from 29 subjects obtained from a study, in which the subjects performed a set of tests based on visual stimuli and motor images of the hands is presented. Three types of data are provided in this article: (1) Signals based on visual events (VEP), (2) signals based on steady state visual events (SSVEP) and (3) signals based upon Motor Imagery (MI). Several research projects have used this data to test the detection of visual stimuli, classification and selection of characteristics of brain signals, EEG preprocessing and for optimization processes based on heuristic algorithms and algorithms based upon collective animal intelligence. The data was acquired using an Emotiv Epoc + portable EEG with 14 data channels and two reference channels.

**Table:** Specifications table

Subject area	*Neuroscience*
More specific subject area	*Neuroinformatics, Multichannel EEG data acquisition*
Type of data	*EEG Raw signals*
How data was acquired	*Portable EEG equipment called EMOTIV, model EPOC+, high-resolution multichannel with 16 electrodes (14 channels with 2 reference channels). Transfer rate is 128 bits per channel; the response frequency is 0.2-*45 Hz *with a resolution of 14-16 bits per channel and a dynamic range of* ± *4.*17mV*. flickering frequencies of 0.*895 Hz*.*
Data format	*Raw data signals, csv file format*
Experimental factors	*The EEG data were acquired during the experimental tests, the subjects of the study were seated in a comfortable chair* 70 cm *away from a standard 15-inch LCD monitor (with a screen resolution of 1024 x 768 and a refresh rate of* 60 Hz*).*
Experimental features	*29 participants made a set of 5 experimental tests: First, visual search of the evoked visual potential (VEP), this test consists of a subject that looks for a small stimulus inside the images of a natural landscape, like a forest, a river, among others. Three different tests named “five box test” for the potential steady state event (SSVEP) is designed to obtain data related to stimuli in the brain signals during a simple attention exercise by looking at a group of five boxes in a screen. Here we seek to find the difference between stimuli related to attention and those not related to attention. Unlike visual tests, the subject must discriminate between different types of stimuli that occur at high speed. Finally, the motor imagery (MI) test consists of showing the images of the subject of the test that resemble any part of the body, with a certain predefined order to better fulfill the purpose of this test.*
Data source location	*Querétaro, México*
Data accessibility	*UCI Machine Learning Repository “EEG Steady-State Visual Evoked Potential Signals Data Set”,*https://archive.ics.uci.edu/ml/datasets/EEG+Steady-State+Visual+Evoked+Potential+Signals#
Related research article	*Fernandez-Fraga S. M., Aceves-Fernandez M. A., Pedraza-Ortega, J. C. (2018). Feature Extraction of EEG Signal upon BCI Systems Based on Steady-State Visual Evoked Potentials Using the Ant Colony Optimization Algorithm. Discrete Dynamics in Nature and Society, 2018.* *S. M. Fernandez-Fraga, M. A. Aceves-Fernandez, J. C. Pedraza-Ortega & J. M. Ramos-Arreguin (2018). Screen Task Experiments for EEG Signals Based on SSVEP Brain Computer Interface. International Journal of Advanced Research, 2018.*

**Table:** 

**Value of the data** • Novelty of the data. Visual Search experiment is based on Dimigen et al. (2011) test [1]. Five box test experiment is based on tests performed visual discrimination by Makeig et al. (1999) [2] and Motor imagery experiments are novel. • The EEG data can be analyzed to investigate the characteristics of brain signals based on visual events as well as stimuli generated by motor images of the hands. • The EEG data has been used for the optimization of signals based on heuristic and probabilistic methods or any machine learning method. • The EEG data can be feature analysis based on the frequency domain using methods of decomposing signals as Fast Fourier Transform (Muller-Putz et al., 2008; Al-Maqtari et al., 2009; Wong et al., 2010; Wang et al., 2010; Diez et al., 2011; Wong et al., 2011; Jia et al., 2011; Punsawad et al., 2012; Hwang et al., 2013; Diez et al.,2013) and Wavelets (Bian et al., 2010; Zhang et al., 2010; Bian et al., 2011. • The EEG data can be feature analysis based on the time domain using fractal dimension methods [3].

## 1 Data

**Fig. 1:**
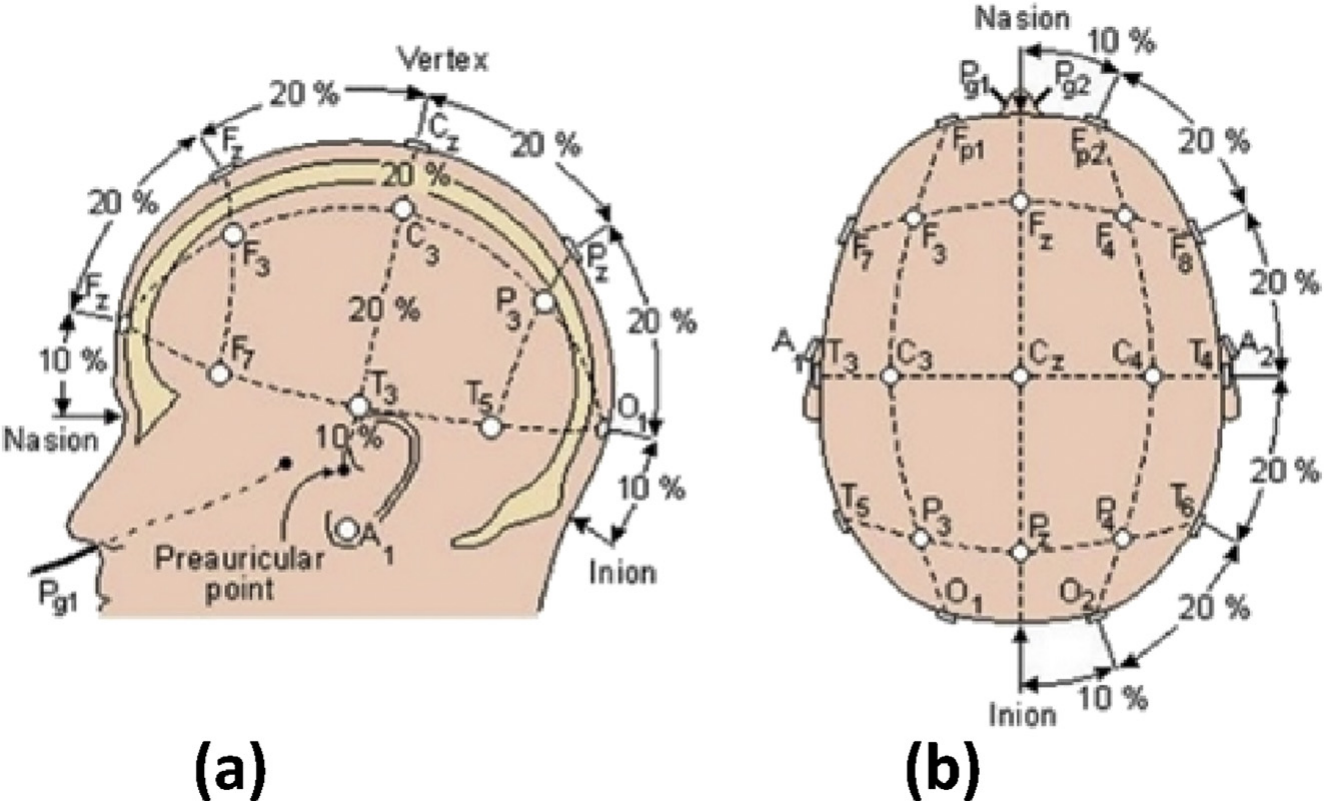
Location of electrodes in the International System of Electroencephalography Societies 10–20. (a) Side view. (b) Top view. (Olivas, 2010).

Twenty-nine healthy volunteers. The EEG signals were obtained by a portable, high resolution multi-brand equipment Emotiv Epoc + EEG model. The equipment has 16 electrodes, 14 data channels and 2 reference channels which were placed in the standard positions of the international 10–20 system (Fig. 1).

The data is presented in csv files format. First row contains information about the data: subject number, date of creation of the data, transfer rate, identification of the subject, identification of the columns, and units. Columns 3–16 contain the information for each of the electrodes based on the international system 10–20: AF3 F7 F3 FC5 T7 P7 O1 O2 P8 T8 FC6 F4 F8 AF4.

## 2 Experimental design, materials, and methods

### 2.1 Statement of ethics

For the development of the evidence contained herein, they were asked all participants to read and sign an informed consent form and written a letter of confidentiality for information management before participating in this study. The study protocol was based on the manual “Ethics of Scientific Research” at the Universidad Autónoma de Querétaro [4].

### 2.2 Research study group

The subjects participating in the elaboration of the tests described were presented voluntarily and declared to be in agreement with the procedure, signed a letter of consent for the realization of the study tests and a letter of confidentiality with reference to their personal data. The testing process does not present any risk. However, it is recommended that study subjects present themselves with a resting period of 6–8 hours, and any subject can suspend the procedure at any time they wish. In the case of a minor, consent to participate in the tests must be authorized and signed by one of their parents or guardians. The data presented are based on a group of subjects, healthy subjects, indistinct sex, indistinct age, regardless of whether they are right-handed or left-handed, with regular vision or with lenses (without eye surgeries), none under medical treatment or taking medication. Twenty-nine healthy volunteers participated, 17 men and 12 women, aged 20–29 and 48–50, participated in the experiment. 17 of them with normal vision and 12 corrected vision.

### 2.3 Risks and inconveniences

The study did not generate risks of any kind for the study subject, because all the verifications of possible electrical risks were made in the data acquisition system, which consists of ten and six non-invasive superficial electrodes for the brain signals acquisition (EEG) placed in specific positions on the scalp. However, minimal risks are not exempted from the subject due to known or foreseeable adverse reactions during the study process. By participating in this research it is possible that the subject experiences some type of minimal discomfort due to the placement of the electrodes or fatigue in front of the visual stimuli. To minimize this type of discomfort, short-term tests were scheduled.

### 2.4 Materials and equipment

To obtain the brain signals, a portable commercially available EEG equipment called EMOTIV, model EPOC+, high-resolution multichannel with 16 electrodes (14 channels with 2 reference channels) were used. Its transfer rate is 128 bits per channel; the response frequency is 0.2–45 Hz with a resolution of 14–16 bits per channel and a dynamic range of ± 4.17mV. The EEG connectivity is 2.4 GHz USB 3.0 wireless and Bluetooth 4.0. The sensor technology is based on a saline hydration medium. The apparatus includes the software Emotiv Xavier Test Bench Software V.3.0.0.22. For the acquisition of brain signals. A laptop with Intel Core i5 processor, 6 GB of RAM, and a 64-bit Windows operating system were used to record the signals. In order to present the tests, a 17″ LCD monitor with a resolution of 1024 × 768 pixels was used. This may vary according to the undergraduate laboratory conditions.

### 2.5 Test set

Cervantes (2008) classifies BCI systems according to signals are acquired in endogenous and exogenous systems [5].

The endogenous systems depend on the ability of the user to control their electrophysiological activity. We can classify endogenous systems in systems based on sensorimotor rhythms (MI by motor imagery), or in systems based on Slow Cortical Potentials (SCP). MI systems, which are, based on the imagery of performing motor actions to evoke signals similar to those observed in actual movement. SCP systems involve slow changes in voltage generated on the cerebral cortex, with a variable duration between 0.5 s and 10 s. and they are typically associated with movement.

Endogenous systems require a period of intensive training.

The exogenous systems are based on the obtaining from evoked related potentials (ERP), depends it on the electrophysiological activity triggered by external stimuli and they do not need an intensive stage of training. They can be classified as evoked potential by P300 events, visual evoked potential (VEP), steady-state visual evoked events (SSVEP) or auditory evoked potential (AEP).

The BCI systems based on P300 usually present to the person a set of stimuli of which only a few have a relationship with the intention of the person. In this way, the stimuli of interest, being not frequent and being mixed with other much more common stimuli. This is due to the appearance of a potential 300 ms after the user realized the brain activity. BCI systems based on VEP and SSVEP are detected on the EEG after a visual stimulus has been applied to the user. BCI systems based on AEP are perceived after seeing the user presenting sources of sounds at different frequencies, the user concentrating on any of them, generates a potential of the same frequency as the stimulus [5]. There are a number of works in which the authors have used the experiments to model the behavior, extract the characteristics of the signals or classify the tests. Some of these works may be further explored in [6], [7], [8].

The current work presents a set of 5 experimental tests: Visual search for VEP, five boxes test for SSVEP (three different tests) and Visual Imagery for MI, which are described below.

### 2.6 Visual Search test

*Summary:* This test consists of a subject looking for a small stimulus inside the images of a natural landscape, such as a forest, river, among others.

*Images:* Natural images in black and white of 800 × 600 pixels are presented in the center of the screen of 1024 × 768 pixels.

*Stimulus:* The stimulus is a yellow circle with a percentage of the screen of 4% of the entire image size that appears on a random position each time.

*Description:* The stimulus appears between 8 and 16 seconds after the start of the image in a random location within the image, the subject must not take any action when the stimulus appears, only locates his position on the screen with his gaze. 1000 ms later, a dark screen with a duration of 3000 ms replaces the image and the test is repeated five times. Because the time needed to show, the stimulus on the screen varies, the time of the test also.

Sequence (Fig. 2):

• Event to synchronize (Dark screen 5 s).
• Show natural image on screen
• Show stimulus in a random location within the image (8–16 s after the image is presented).
• Show dark screen (1000 ms later)
• Repeat the experiment five times.

**Fig. 2:**
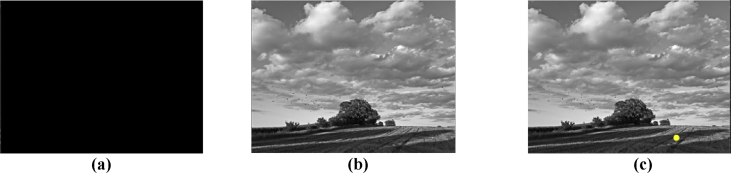
Visual Search Test Setup. (a) Wait screen, Initial screen. (b) Grayscale image. (c) Stimulation within the image.

### 2.7 Five box test

*Summary:* It's designed to obtain data related to stimuli in brain signals during a simple attention exercise by staring a group of five boxes on a screen. Here we seek to find the difference between stimuli related to attention and those not related to attention. Unlike visual tests, the subject must discriminate between different types of stimuli that are presented at high speed.

*Stimuli:* The stimuli that occur within this box are stimuli attended. Stimuli inside the blue boxes. are known as unattended stimuli. All tests contain 100 stimuli in total; 80 are not attended and.

20 must be attended. The stimuli are presented to the subject in a random position at 250 ms, 500 ms, 750 ms, and 1 s. The stimuli lasts 200 ms on the screen before disappearing*.*

*Test* variations:

• Simple Discrimination: The stimuli are red circles within the 5 boxes. 120 (25%) events attended and 480 (75%) events not attended.
• Composite Discrimination: The stimuli are boxes filled with their original color. 120 (25%) events attended and 480 (75%) events not attended.
• Combined Discrimination: Test considers both previous tests. Half of the stimuli are of simple variant and the other half of composite variant. The stimuli attended are only the red circles. 35 (25%) events attended and 105 (75%) events not attended.

**Fig. 3:**
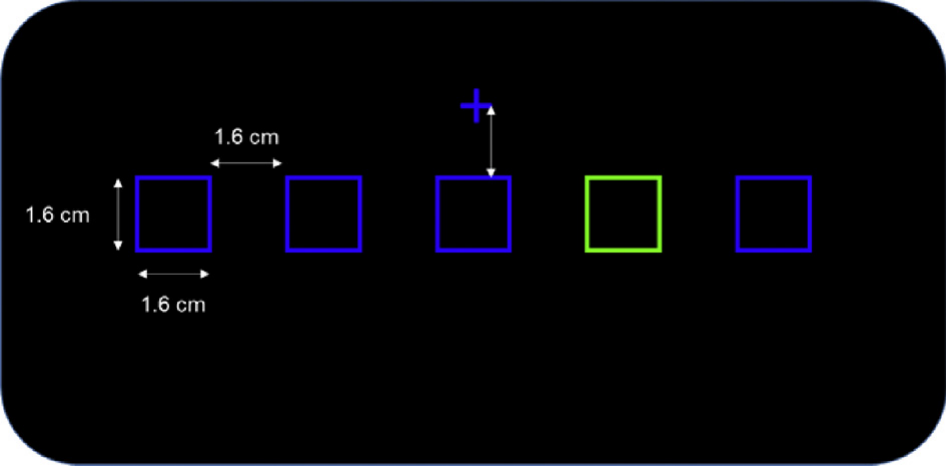
Five box test configuration.

*Description of the Test Screen:* In the test the users fix their eyes on a cross, above which five boxes (boxes) were constantly displayed (Fig. 3). Each test block is 76 s, one of the boxes was different color (green). The location of this table was randomized during the testing periods. A series of circles were briefly presented in any of the five boxes in a random order. The user was asked to concentrate each time a disc appeared in one of the frames recorded from subjects who attended the random sequences of full discs appear briefly inside one of the five empty squares that were shown (Townsend and Courchesne 1994). The 1.6 cm (0.63 in) square contours are shown on a black background at horizontal visual angles of 0° ± 2.7° and 5.5° ± of fixation. During each block of 76 s of the trials, one of the five general lines was green and the other four blue.

**Fig. 4:**
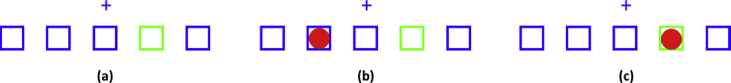
Test 5 boxes setup. (a) Initial configuration. (b) Unattended stimulus.(c) Assisted stimulus (event).

The green square marked the location they will attend. This location was varied in order to randomly counterbalance through blocks. In each block, 100 stimuli (full red discs) are displayed for 117 ms in one of the five empty squares in a pseudo-random sequence with inter-stimulus intervals (ISIS) of 250 ms, 500 ms, 750 ms and 1 s. In each test 100 stimuli are presented, 20 attended (events) 80 unattended (Fig. 4b and c respectively), the last stimulus of 200 ms remains on the screen before disappearing [9].

### 2.8 Motor imagery test

*Summary:* The motor imagery test consists of showing the test subject images that resemble any part of the body, with certain predefined order to best fulfill the purpose of this test.

*Images:* Artificial color images of 800 × 600 pixels are presented in the center of the 1366 × 768 pixels screen.

*Stimulus:* The stimuli used are digital representation human hands; each stimulus has a color code to facilitate identification by the test subject. The open hands are red and the closed hands are blue.

*Description:* The purpose of the test is that when observing the image shown, the test subject generates a mental image of the part of the body that represents the image and, in doing so, generates a brain impulse, which can be acquired and registered by the acquisition system.

To further facilitate the identification of the images by the test subject, in the center of the test there is a cross in which the view of the subject is centralized and to the sides (correspondingly with the hands) the images of the extremities appear, all on a completely white background.

Each event (motor imagery shown) lasts 5 seconds on the screen; this provides a framework for reaction and capture of the brain signal. The complete test lasts 30 seconds.

**Fig. 5:**
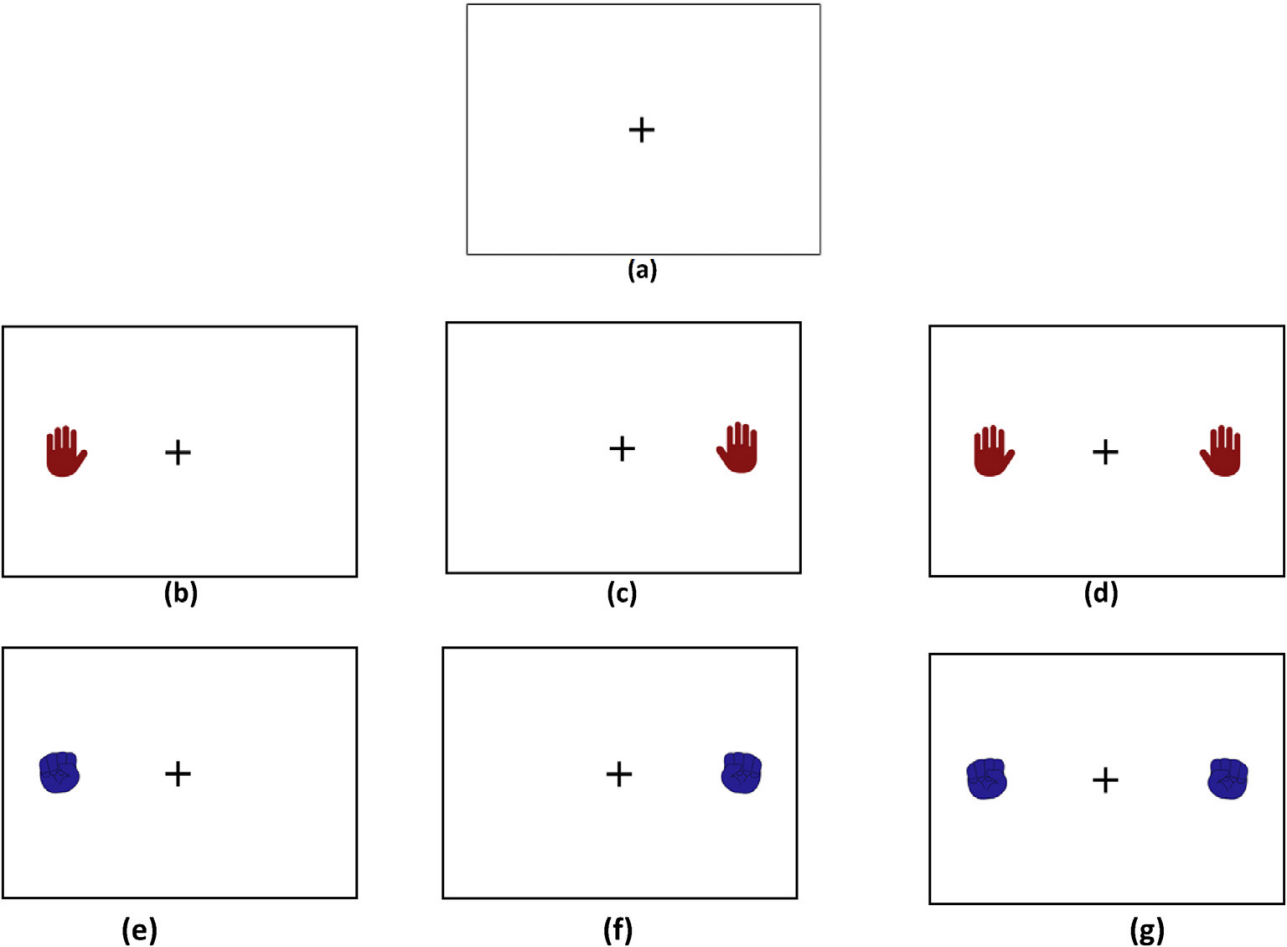
Hand Shake Test setup. (a) Stabilization screen. (b) Open right hand screen. (c) Open left hand screen. (d) Screen both open hands. (e) Left hand screen closed. (f) Right hand screen closed. (g) Screen both hands closed.

Sequence (Fig. 5):

• Stabilization image (Fig. 5a).
• Left hand open (Fig. 5b).
• Right hand open (Fig. 5c).
• Both hands open (Fig. 5d).
• Left hand closed (Fig. 5e).
• Right hand closed (Fig. 5f).
• Both hands closed (Fig. 5g).

### 2.9 Experiment procedure

In order to show the protocol for the experimentation process, the steps for the development of the tests described above are as follows:

1. The general procedure of the testing process is explained to the possible volunteer in a clear and precise manner; as well as the test subject will be given the opportunity to ask questions which must be answered by the students involved before the test commences. This will be overseeing by the teacher and an expert physician at all times. If the volunteer and/or the tutor (in case of a minor) of the volunteer decide not to continue with the test, the session will be terminated.
2. If the volunteer and/or responsible for the volunteer decide to continue with the test, they must sign the consent letter, the confidentiality letter and then the data registration form is filled out (all of them in their original language).
3. To start the tests the first thing that must be done is to organize and prepare the materials and equipment to be used. This will help the students to fully understand the equipment, material, and the letter's content, which is part of their learning process.
4. Ensure that the electroencephalogram is on proper working conditions and the student's handbook provided in class. This will include, connecting and charging the equipment for at least 4 hours prior to the experiments take place, hydrate the electrodes with saline solution to ensure proper electrical conductivity (a couple of drops of saline solution may be sufficient for each electrode), assembling the electrodes in the equipment once they are properly soaked in the saline solution.
5. The students also have to get involved in the device connection. This includes the USB transceiver, testing the communication between the computer program that acquired the signals with the EEG device, assuring that the device is well placed and the electrodes are reading a signal from the subject's head prior to initiate the experiments. Also, the students must check that each electrode is making contact with the scalp and the electrodes are placed according to the international standard 10–20.
6. Once the subject has the equipment on, and sited on a chair, which should be at most 700 mm (27.5 inch) approximately of the screen that is, generating the stimulus, ensuring that it is as comfortable as possible since this will allow a better comfort during the test.
7. The students involved will proceed to explain the test at hand every time the test is about to start. This procedure will repeat until the tests are over. After all the tests are completed or the subject decides not to continue any longer, the electroencephalograph device will be removed from the subject's head.

### 2.10 Identification of data files

Table 1 shows the identification format of the files generated by the experiments; the system experiment generates SCV format electroencephalogram signal file.

**Table 1:** Identification format file.

GROUP	ID SUBJECT			DATA TYPE	STUDY TYPE	SUB-TYPE TRIAL	TRIAL INSTANCE		FILE EXTENSION
A	0	0	1	S	M	1	_	1	.csv
									

Fields Description.

(a) Group: A → Healthy people.

(b) Subject ID: Registration Number.

(c) Data Type: E → Events, S → Signals.

(d) Study Type: V → Visual Search, M → Hand Shake, B → Five Box.

(e) Sub-type trial: (specifically for 5 box test) 1 → simple discrimination, 2 → compound discrimination, 3 → combined discrimination.

(f) Trial instance: number of times the test is repeated.

(g) File extension: csv.

Data base field's description.

Table 2 and Table 3 show the format in which subject information and test results obtained will be presented respectively.

**Table 2:** Subject information.

Register No.	SUBJECT INFORMATION										
	GROUP	GENDER	AGE	HANDED CONDITION	HEALTHY	GLASSES	SMOKE	MEDICAL TREATMENT	MEDICINES	SLEEP HRS	FASTING HRS
1	A	MALE	48	LEFT	YES	YES	NO	YES	YES	6	2

**Table 3:** Test information.

TEST INFORMATION								
TRANSFER RATE (bps)	TEST TYPE	TEST NAME	EVENTS No.	EVENT TIME (ms)	TRIAL DATA	DATA REGISTER	STABILIZATION TIME (sec)	TEST DURATION (sec)
128	VISUAL	IMAGE SEARCH	5	250	A001SV1_1.EDF	A001SV1_1.PDF	5	96

Fields Description.

1. Register No.: Study subject Number id.

2. Group: Group id (A/B).

3. Gender; Male/Female.

4. Age: subject age.

5. Handed Condition: Left/Right.

6. Healthy: The subject is a healthy person or disability person. Yes/No status.

7. Glasses: Visual condition subject. Yes/No status.

8. Smoke: Subject condition. Yes/No status.

9. Medical treatment: Subject condition about his health before the experiment. Yes/No status.

10. Medicine: Subject uses medications before the experiment. Yes/No status.

11. Sleep: Sleeps number of hours before the experiment.

12. Fasting: Fasting number of hours before the experiment.

13. Transfer Rate: Electroencephalogram information transfer rate in bits/sec.

14. Test Type: Visual or Motor Image test.

15. Test Name: Visual Search, Five Box 1, Five Box 2, Five Box 3, Hand Shake.

16. Events No.: test events number.

17. Event Time: event duration in milliseconds.

18. Trial Data: file identification name corresponds to test signals.

19. Data Register: file identification name corresponds to EEG data register.

20. Stabilization Time: duration of the user's stabilization time before beginning the test in seconds.

21. Test Duration: test duration including stabilization time.
