# Supplementary material for: EEG data collection using visual evoked, steady state visual evoked and motor image task, designed to brain computer interfaces (BCI) development
Source: Data Brief. 2019 Mar 22;25:103871. doi: 10.1016/j.dib.2019.103871 (PMC6581785; doi:10.1016/j.dib.2019.103871)
Supplement: Multimedia component 1 [file mmc1.docx]

Conflict of Interest and Authorship Conformation Form

Please check the following as appropriate:

- All authors have participated in (a) conception and design, or analysis and interpretation of the data; (b) drafting the article or revising it critically for important intellectual content; and (c) approval of the final version.
- This manuscript has not been submitted to, nor is under review at, another journal or other publishing venue.
- The authors have no affiliation with any organization with a direct or indirect financial interest in the subject matter discussed in the manuscript
- The following authors have affiliations with organizations with direct or indirect financial interest in the subject matter discussed in the manuscript:

Author’s name Affiliation

Santiago Fernandez Fraga Tecnológico Nacional de México/

Instituto Tecnológico de Querétaro

Marco Antonio Aceves Fernandez Universidad Autónoma de Querétaro

Jose Carlos Pedraza Ortega Universidad Autónoma de Querétaro
